# Family physicians as advocates for primary health care in Botswana

**DOI:** 10.4102/phcfm.v17i1.4908

**Published:** 2025-04-30

**Authors:** Billy M. Tsima, Yaone Bogatsu, Keneilwe Motlhatlhedi, Claire Brockbank, Sunanda C. Ray

**Affiliations:** 1 Department of Family Medicine and Public Health, Faculty of Medicine, University of Botswana, Gaborone, Botswana; 2 Botswana Association of Family Physicians, Gaborone, Botswana; 3 Department of Medical Education, Faculty of Medicine, University of Botswana, Gaborone, Botswana

**Keywords:** advocacy, family physicians, primary health care, Botswana, health system strengthening

## Abstract

Advocacy for family medicine in Botswana has been facilitated by good cooperation between the University of Botswana (UB), the Botswana Association of Family Physicians and the Botswana Health Professions Council. The importance of family physician leadership in putting primary health care (PHC) at the centre of the health system has been recognised and acted on by the Ministry of Health. Family medicine teaching is integrated throughout the 5-year undergraduate medical curriculum at the UB Faculty of Medicine and takes place in rural and urban training complexes. Family medicine is a popular career choice but developing a critical mass of family physicians is limited by the low number of training posts available each year. Botswana has a strong PHC foundation with village health committees, village development committees and community home-based care teams, led by district health management teams (DHMTs). There are not enough family physicians currently in the public sector to provide sufficient clinical leadership for the DHMTs as well as to provide clinical supervision of medical officers and nursing staff in clinics, and primary and district hospitals, as well to be actively involved in advocacy for health equity and quality of care to policymakers. Active participation in regional initiatives to expand family physician training opportunities could contribute to strengthening the health workforce in Botswana.

## Introduction

Advocacy for the integration of family medicine (FM) into healthcare systems, requires strategic efforts to raise awareness of the discipline, influence policies, and foster support for the recognition and development of FM, advocacy for universal health coverage and to address healthcare challenges such as access, equity, and sustainability. The tactical approach used by the University of Botswana (UB) began with a workshop in 2008 to explore the demand for family physician (FP) specialists in Botswana. It was attended by representatives from the Ministries of Health, Education and Local Government, the Botswana Health Professions Council (BHPC) and international FM advocates including Professor Mash of Stellenbosch University, South Africa. Following this workshop, the BHPC recognised FM as a speciality and the Integrated Health Services Plan included FM as a critical speciality for the first time in 2011.^1^ The postgraduate specialist training programme in FM Master of Medicine (MMed) was launched at UB with a first intake of trainees in 2011 rotating through district hospital training centres. The South-South academic partnership with Stellenbosch University with curriculum and assessment support, has been vital for recognition and sustainability. The first FPs graduated in 2016. By 2024, 40 FPs had graduated with MMed FM: 29 are working in the public sector, four in academia, four in private mine hospitals, and three in full time private practice, so the majority have been retained as a public resource.

## Family medicine training at University of Botswana

Some countries have struggled to recruit trainees to their postgraduate specialist FM training programmes because of limited understanding of what FM is.^2,3^ One way to familiarise medical graduates to FM as a career option is through engaging them as medical students at an early stage, by integrating FM teaching all through the undergraduate curricula and early exposure to people presenting with illness in primary care.^2,4,5^ The UB Faculty of Medicine (UBFoM) developed such an integrated programme when the undergraduate medical (MBBS) programme was launched in 2009.^1^ Medical students undertake 8 weeks of FM in year 3 and 8 weeks in year 5 of their 5-year MBBS programme, as well as primary care visits in 1st and 2nd year, which includes clinical and communication skills teaching in urban municipality clinics.

As a result, there is a consistently high number of applicants for the 4–6 training positions offered by the UBFoM FM MMed programme each year. For FM to demonstrate impact on health outcomes for the whole country rather than mainly on urban centres, a strategic distribution of FPs to supervise clinical care in district and primary care facilities is necessary. In Botswana, primary hospitals provide care for most diseases, injuries and emergencies while referring complex cases to district and central hospitals. The seven district hospitals function more like provincial hospitals in other African countries, with a larger number of beds (150–270) and posts for specialist clinicians including FPs. They manage higher level inpatient and outpatient services, emergency and urgent care as well as chronic conditions and long-term conditions.^i^ Botswana has two main district hospital-based FM training centres, one in Maun (northern Botswana) and Mahalapye (central Botswana).^1^ The UB Department of FM and Public Health has demonstrated a leadership role in defining attributes of resilient healthcare systems through collaborative operational and implementation research and publications on aspects related to service delivery, disease patterns and management of conditions prevalent in Botswana. The Department has contributed to more than 75 publications in international journals (for example^6,7,8)^ and played a significant role in reorganising services and training during the coronavirus disease 2019 (COVID-19) pandemic including using telehealth and distance learning methods.^9,10^

## Relationship with Ministry of Health

Botswana has a population of 2.3 million with 67% living in urban areas (2022 census). This population is served by three referral, seven district and 18 primary hospitals. Ideally each primary hospital and its surrounding clinics (serving a population of 1–200 000) should have at least one FP overseeing the clinical services provided by medical officers, nurse practitioners and other allied health professionals, as well as providing outreach supervision to smaller peripheral clinics. Family physicians play an important role heading some of the 18 district health management teams (DHMTs) in the country, filling critical FP roles of manager (management of facilities and the district, especially clinical governance and improving quality of care) and leading a community-orientation in health services (responding to public health issues arising from the community served, coordinating with public health specialists, other governmental sectors, community leaders and non-governmental organisations).^2^ All seven district hospitals and 8 of the 18 primary hospitals have an FP in place. The Government of Botswana has advocated for an integrated and coordinated patient-centred healthcare model that aligns with primary health care (PHC) principles of comprehensive and sustainable healthcare.^11^ Family physicians are well-positioned in identifying how to achieve this.

Botswana has a strong PHC foundation based on village health committees, village development committees and community home-based care teams. There are also district health structures and sub-committees, which are part of the local administration and which DHMTs lead. Primary care facilities were moved to the Ministry of Health (MoH) in 2010 from the Ministry of Local Government, and are now going back to Ministry of Local Government (MoLG) as part of a restructuring of health services in which primary care becomes the centre of the health sector, with FPs taking the lead. It therefore becomes crucial that the role of FPs be emphasised not only at primary and district hospitals but as leaders of primary care teams in local government. This will necessitate an increase in numbers and acknowledgement of the status of FPs as leaders. Clarification of roles and responsibilities in relation to public health specialists will also be essential. These changes provide an excellent opportunity for Botswana to work towards community orientated primary care (COPC). This is a continuous process by which PHC is provided to a defined community on the basis of its assessed health needs, by the planned integration of primary care practice and public health.^12,13^ This model works to identify specific health needs and disparities within communities, their relationship with social determinants of health, and tailor interventions accordingly. A shortcoming is that there are not enough FPs currently in the public sector to provide sufficient clinical leadership for the DHMTs as well as to provide clinical supervision of medical officers and nursing staff in clinics, and primary and district hospitals, as well to be actively involved in advocacy for PHC and COPC to policymakers.

## Scope of practice

The scope of practice (SOP) for a health professional is defined as the limits of their knowledge, skills and experience and is made up of the tasks and activities carried out within their professional roles. It is informed by the professionals’ training and experience. The SOP concept enables health regulators, leaders and managers to ensure safe practice and for FPs that their skills development is in keeping with the expectations for their speciality.^14^ The SOP for FPs in Botswana was formulated at a BHPC SOP Workshop in 2015, with representation from public and private sector FPs as well as academics from UBFoM.

Family physicians practise in diverse primary care settings, requiring practical skills appropriate to those settings. A requirement was made that all FPs should have adequate proficiency in at least 80% of the skills listed within the SOP, which were based on skills specified in the UB FM MMed training handbook. Continuing Medical Education requirements for continued registration with the BHPC were also stated, including a mandatory Basic Life Support qualification to be renewed every 5 years. The collaborative development of the SOP facilitated confidence within the medical community of the competency of FPs and clearly demarcated the difference between FPs and non-specialist general practitioners. It was reinforced by the principle that SOP decisions should be responsive to population health needs and benefit rather than to professional self-interest.

## Partnership with Botswana Association of Family Physicians

Professional associations play a key role in promoting the status and importance of a medical speciality, influencing healthcare policy, and advancing the interests of their members. The Botswana Association of FPs (BAOFP) partners closely with FM academics and jointly advocate for FM and PHC. An example is production of evidence-based clinical guidelines, which have been developed collectively for use in the public sector. The BAOFP and the Department of FM and Public Health (together with the Botswana Association of Family Nurse Practitioners) host the Botswana National Family Medicine Annual Conference (now in its 10th year) where they showcase FM as a discipline and advocate for coordination in primary care. Botswana Association of Family Physicians recently won the bid to host the 2026 WONCA Africa regional conference, which will also raise the profile of FM in Botswana and the region. These conferences provide excellent platforms for educating policymakers and healthcare leaders about the value of FM in improving health outcomes for the population. They also provide opportunities to work with various media to highlight success stories and evidence of how FM in Botswana contributes to greater equity and improved quality of healthcare. Because of its critical mass of senior FPs, BAOFP has been able to take a lead role in the recent establishment of the East Central and Southern Africa College of Family Physicians (ECSA-CFP), advising on curriculum development, accreditation processes and mobilisation of members.

## Conclusion

### Lessons learnt and the way forward

The successful development of FM as a recognised and valued speciality in Botswana can be attributed to strategic partnerships between academic institutions, the MoH and professional associations. A key aspect has been to demonstrate the depth of expertise and wide-ranging skills FPs work with despite being considered ‘generalists’. Showcasing our achievements is performed through presentations of our work at workshops and conferences, and publications in partnership with other specialists at UBFoM. One FP role is to identify unmet need in communities for more complex care, for instance in surgery and non-communicable diseases for referral for more specialist care, and for providing continuity of care for these individuals on discharge. Such integration requires close collaboration with public health colleagues, such as through DHMTs, using routine health data to pattern disease epidemiology and responses, which has seen some success with communicable and non-communicable disease programmes. Members of the FP team are well-placed in various advisory committees at MoH and the UBFoM ensuring that FM always has a presence on the agenda. This has helped us to raise the profile of FM nationally and regionally.

In most African countries, FPs are a scarce resource and do not provide first-contact primary care except in private practice. They are usually based in district or primary hospitals, as clinicians for complex cases, supervisors and consultants to multidisciplinary PHC teams, educators and capacity builders and leaders of clinical governance.^15^ This is true also of Botswana. To make a significant impact on health outcomes for the whole country, to deliver high-quality, reliable frontline healthcare within local communities, the training of FPs in Botswana needs considerable expansion to increase numbers, so that FPs can indeed become the cornerstone of comprehensive PHC, as described internationally.^16,17^ This imperative of a critical mass is more urgent than for other specialists, because FPs function at the intersection of clinical practice and community engagement, championing patients’ rights, influencing healthcare policies and addressing disparities in underserved communities. These are arguments that FP academics, associations and practitioners can make to justify greater investment in FM, to make the whole health system more efficient and promoting comprehensive sustainable health programmes. The participation of BAOFP and UBFoM in training and examinations through the ECSA-CFP will provide opportunities for and achieving higher FP numbers in Botswana as well as sharing resources and supporting such training for the whole ECSA region.^18^

The author, S.C.R., serves as an editorial board member of this journal. S.C.R. has no other competing interests to declare.

## Footnotes

i
www.moh.gov.bw/hospitals1.html
